# The Genotoxin Colibactin Shapes Gut Microbiota in Mice

**DOI:** 10.1128/mSphere.00589-20

**Published:** 2020-07-01

**Authors:** Sophie Tronnet, Pauline Floch, Laetitia Lucarelli, Deborah Gaillard, Patricia Martin, Matteo Serino, Eric Oswald

**Affiliations:** a IRSD, Université de Toulouse, INSERM, INRAE, ENVT, UPS, Toulouse, France; b CHU Toulouse, Hôpital Purpan, Service de Bactériologie-Hygiène, Toulouse, France; University of Michigan—Ann Arbor

**Keywords:** colibactin, enterobacteria, gut microbiota dysbiosis, microbiome

## Abstract

The genotoxin colibactin produced by resident bacteria of the gut microbiota may have tumorigenic effect by inducing DNA double-strand breaks in host cells. Yet, the effect of colibactin on gut microbiota composition and functions remains unknown. To address this point, we designed an experiment in which pregnant mice were colonized with the following: (i) a commensal Escherichia coli strain, (ii) a commensal E. coli strain plus a genotoxic E. coli strain, (iii) a commensal E. coli strain plus a nongenotoxic E. coli mutant strain unable to produce mature colibactin. Then, we analyzed the gut microbiota in pups at day 15 and day 35 after birth. At day 15, mice that were colonized at birth with the genotoxic strain showed lower levels of *Proteobacteria* and taxa belonging to the *Proteobacteria*, a modest effect on overall microbial diversity, and no effect on gut microbiome. At day 35, mice that received the genotoxic strain showed lower *Firmicutes* and taxa belonging to the *Firmicutes*, together with a strong effect on overall microbial diversity and higher microbial functions related to DNA repair. Moreover, the genotoxic strain strongly affected gut microbial diversity evolution of pups receiving the genotoxic strain between day 15 and day 35. Our data show that colibactin, beyond targeting the host, may also exert its genotoxic effect on the gut microbiota.

**IMPORTANCE** Infections of genotoxic Escherichia coli spread concomitantly with urbanized progression. These bacteria may prompt cell senescence and affect DNA stability, inducing cancer via the production of colibactin, a genotoxin shown capable of affecting host DNA in eukaryotic cells. In this study, we show that the action of colibactin may also be directed against other bacteria of the gut microbiota in which genotoxic E. coli bacteria have been introduced. Indeed, the presence of genotoxic E. coli induced a change in both the structure and function of the gut microbiota. Our data indicate that genotoxic E. coli may use colibactin to compete for gut niche utilization.

## Abstract (precis)

Infections of genotoxic Escherichia coli spread concomitantly with urbanized progression. These bacteria may prompt cell senescence and affect DNA stability, inducing cancer via the production of colibactin, a genotoxin shown capable of affecting host DNA in eukaryotic cells. In this study, we show that the action of colibactin may also be directed against other bacteria of the gut microbiota in which genotoxic E. coli bacteria have been introduced. Indeed, the presence of genotoxic E. coli induced a change in both the structure and function of the gut microbiota. Our data indicate that genotoxic E. coli may use colibactin to compete for gut niche utilization.

## INTRODUCTION

The quantitative and qualitative alterations, named dysbiosis, of the gut microbiota are now considered key traits of multiple pathologies such as metabolic, inflammatory, and infectious diseases (1). Dysbioses can originate from several factors related to multiple habits with antibiotic abuse being among the most important. However, an increased fat-to-fiber alimentary ratio, which is a common inducer of metabolic diseases, represents the strongest trigger of gut microbiota alterations. In this context, it was shown that a high-fat/high-sucrose diet was able to increase the intestinal adherence of adherent invasive Escherichia coli (AIEC) in mice, thus promoting AIEC infection (2). Both proinflammatory and genotoxic strains belong to E. coli, which is a dominant member of the phylum *Proteobacteria* and colonizes the guts of both humans and animals at birth (3). Only 40% of the whole genome of E. coli is conserved, which confers a huge molecular plasticity to these bacteria. The plasticity is characterized by the acquisition of mobile elements such as plasmids, transposons, phages, and pathogenicity islands (4). Genotoxic E. coli bacteria harbor in their genome a 52-kb polyketide synthase (*pks*) pathogenicity island with genes encoding a complex enzymatic machinery synthesizing the genotoxin colibactin. The B2 phylogenetic group of E. coli is the group harboring the most (30%) of *pks*^+^ E. coli strains. The prevalence of the B2 group increased dramatically in the last decades and concomitantly with the progression of urbanization (5) and both autoimmune and allergic diseases (6). This event induced a progressive passage from phylogenetic group A (with no *pks*^+^ E. coli strains) to phylogroup B2. Colibactin is capable of inducing DNA double-strand breaks in eukaryotic cells (7) and of generating DNA interstrand cross-links (8) and was shown to induce multiple alterations in the host such as cell senescence (9, 10), increased E. coli-induced lymphopenia (11), altered intestinal homeostasis (12), modified tumor microenvironment (13), and colon tumor growth (9). Yet, whether colibactin may exert its genotoxic effect even on members of the gut microbiota remains unknown. To address this point, we set up a model of mother-to-pup vertical transmission of both genotoxic and nongenotoxic E. coli, since these strains are already present at birth (3). We studied the effects on both gut microbiota and microbiome on pups at day 15 and 35 after birth. Beyond colibactin, *pks* island genes are involved in the synthesis of multiple factors such as bacterial analgesic lipopeptide (14) and antibiotic molecules such as siderophore-microcins (15). Given the strong importance of the mother as an E. coli transmitting factor (16), to obtain mouse pups colonized by E. coli the most natural way, we decided to colonize the mother with the E. coli of interest. Therefore, to ascribe to the sole production of colibactin the putative effects on gut microbiota, we decided not to compare a *pks*^+^ versus a *pks* mutant E. coli strain but, rather, to generate two isogenic bacterial mutants on the basis of the E. coli SP15 strain (11) and to colonize the mother with these mutants.

## RESULTS

### Colibactin targets the gut microbiota at the onset of intestinal colonization by genotoxic E. coli.

**FIG 1:**
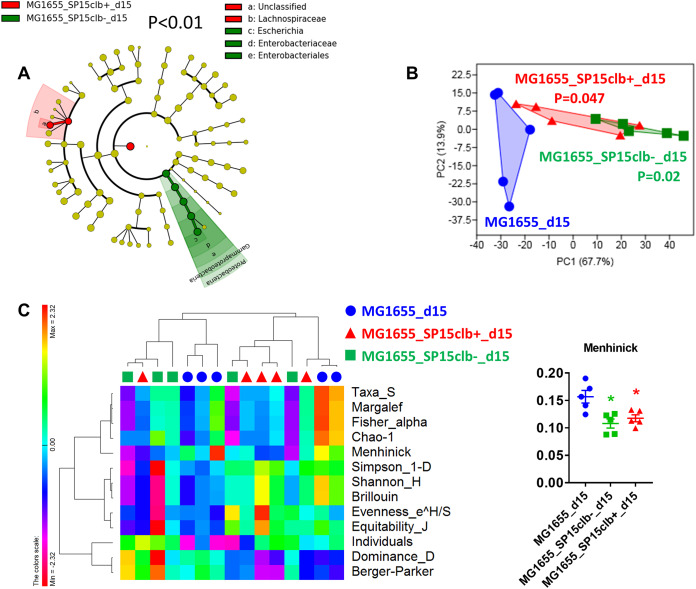
Cecum microbiota of mice aged 15 days following colonization with genotoxic or nongenotoxic E. coli. (A) Cladogram showing bacterial taxa significantly (*P* < 0.01) higher in the group of mice of the same color, according to the enterobacterial strain received at birth. The cladogram shows the taxonomic levels represented by rings with phyla at the innermost ring and genera/species at the outermost ring, and each circle is a bacterial member within that level. All groups of mice have been analyzed, but the cladogram does not show the control group MG1655_d15, meaning this group is not characterized by any higher bacterial taxon compared to the other groups. (B) Principal-component analysis (PCA) showing cluster of groups according to the cecum microbiota (following the color code of the overall figure). The *P* values compared to the value for the control group (blue) are shown. (C) Heat-map based on Pearson distance and complete linkage drawn with the PermutMatrix software (29) showing multiple diversity indices and dot-plot representation of the Menhinick index on the right side (*n* = 5).*, *P* < 0.05 versus control group, Kruskal-Wallis test followed by a two-stage linear step-up procedure of Benjamini, Krieger, and Yekutieli to correct for multiple comparisons by controlling the false-discovery rate (<0.05).

**FIG 2:**
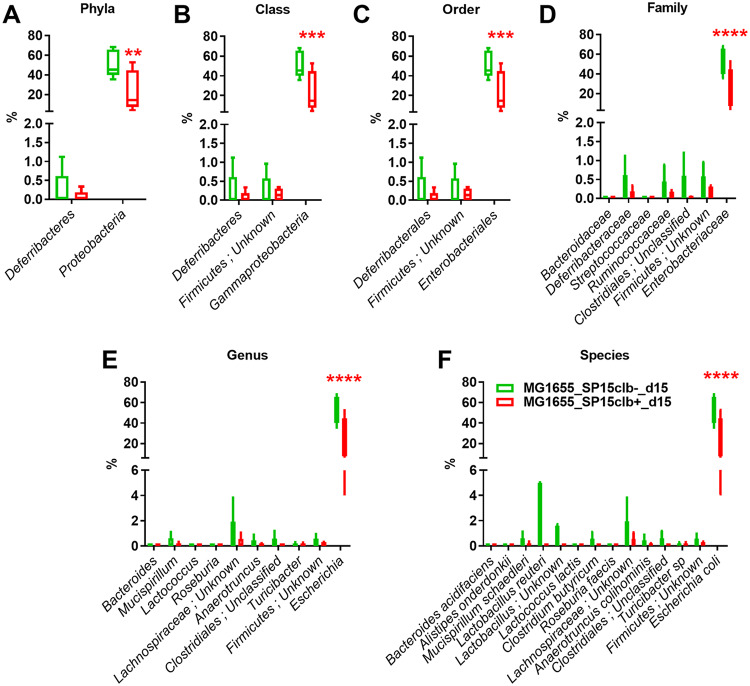
Bacterial taxon reduction following colonization at day 15 with genotoxic or nongenotoxic E. coli. (A to F) Phylum to species histogram representation. *n* = 5. **, *P* < 0.01, ***, *P* < 0.001, ****, *P* < 0.0001, two-way ANOVA followed by a two-stage linear step-up procedure of Benjamini, Krieger, and Yekutieli to correct for multiple comparisons by controlling the false-discovery rate (<0.05).

**FIG 3:**
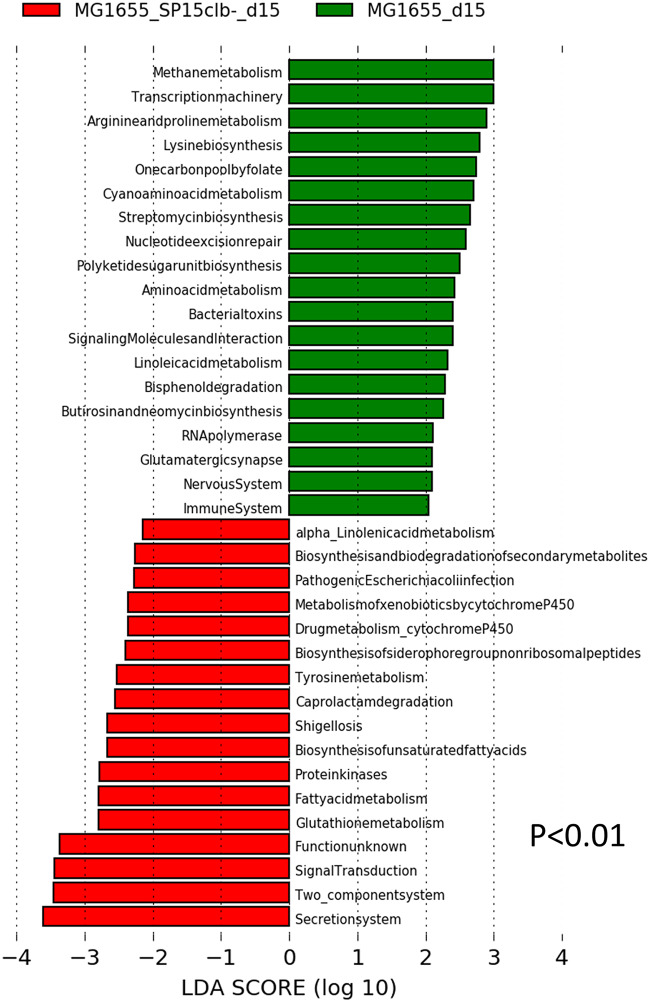
Cecum microbiome of mice aged 15 days following colonization with genotoxic or nongenotoxic E. coli. List of the bacterial functions enriched in the control group MG1655_d15 (green) or in the MG1655_SP15clb-_d15 group (red) and the related linear discriminant analysis (LDA) score (*P* < 0.01). *n* = 5. (All groups of mice have been analyzed, but the figure does not show the MG1655_SP15clb+_d15 group, meaning this group is not characterized by any higher bacterial taxon compared to the other groups).

To understand whether the genotoxin colibactin may also target the host gut microbiota beyond the effects observed on host cells (7, 8), we applied a protocol of vertical mother-to-pup transmission (see Fig. S1 in the supplemental material). Briefly, pregnant mothers were given by gavage either a nongenotoxic E. coli commensal strain (MG1655 strain, phylogroup A, control group) or both MG1655 and the genotoxic E. coli SP15 strain (phylogroup B2, SP15clb+) or its nongenotoxic mutant (SP15clb-). The molecular strategy applied to generate the two isogenic E. coli SP15 strains is reported in Fig. S2. We analyzed the overall putative changes in the gut microbiota of mouse pups at 15 days after birth. The mother-to-pup transfer of the nongenotoxic E. coli SP15clb- strain was associated with a higher relative abundance of *Proteobacteria*. In contrast, the mother-to-pup transfer of the genotoxic E. coli SP15clb+ strain was associated with a higher relative abundance of the family *Lachnospiraceae* (Fig. 1A). Both the nongenotoxic and the genotoxic E. coli SP15 strains significantly affected the overall gut microbiota profile (Fig. 1B). However, the overall gut microbiota diversity was unaffected, despite the significant reduction in the Menhinick index, regardless of the genotoxicity of the E. coli SP15 strain (Fig. 1C). Then, considering the putative antibiotic activity of colibactin (17), we focused on microbial taxa whose abundance was lower after colonization with the genotoxic E. coli SP15 strain. As reported in Fig. 2A to F, the phylum *Proteobacteria* and all the other related taxa displayed a significantly lower abundance in mice colonized with the genotoxic E. coli SP15clb+ strain compared to the nongenotoxic E. coli SP15clb- strain. Next, we analyzed the gut microbiome by performing a functional analysis based on PICRUSt (18). As reported in Fig. 3, we identified microbial pathways significantly enriched in the control MG1655_d15 and in the group of mouse pups cocolonized with the nongenotoxic E. coli SP15clb- strain, but not with the group cocolonized with the genotoxic E. coli SP15clb+ strain. Overall, these data show that 15 days after birth, E. coli genotoxic activity exerts an intraspecies taxonomical but not functional impact on the gut microbiota.

10.1128/mSphere.00589-20.1 FIG S1 Time line of the experimental protocol employed in the cocolonization model of vertical mother-to-pup transmission. Pregnant Swiss CD1 mothers were received and treated with 5 mg/ml of streptomycin from day −3 to day 0 (birth of pups). At day −1, the pregnant Swiss CD1 mothers were given 10^9^ CFU of the enterobacterial strain or a mix of strains (5 × 10^8^ CFU per strain) by gavage: MG1655 (commensal E. coli strain), genotoxic E. coli SP15clb+ strain, or nongenotoxic E. coli SP15clb- strain. All the strains employed in this study are streptomycin resistant. The cecum microbiota was sampled and analyzed on day 15 and day 35 after birth. Download FIG S1, TIF file, 0.5 MB. Copyright © 2020 Tronnet et al. 2020 Tronnet et al. This content is distributed under the terms of the Creative Commons Attribution 4.0 International license.

10.1128/mSphere.00589-20.2 FIG S2 Molecular strategy used to generate the nongenotoxic and genotoxic E. coli SP15 strains. Molecular details of the pCM17 plasmid used to generate the two isogenic mutants of E. coli SP15 strains. Download FIG S2, TIF file, 1.1 MB. Copyright © 2020 Tronnet et al. 2020 Tronnet et al. This content is distributed under the terms of the Creative Commons Attribution 4.0 International license.

### Colibactin targets the gut microbiota and microbiome following intestinal colonization by genotoxic E. coli.

**FIG 4:**
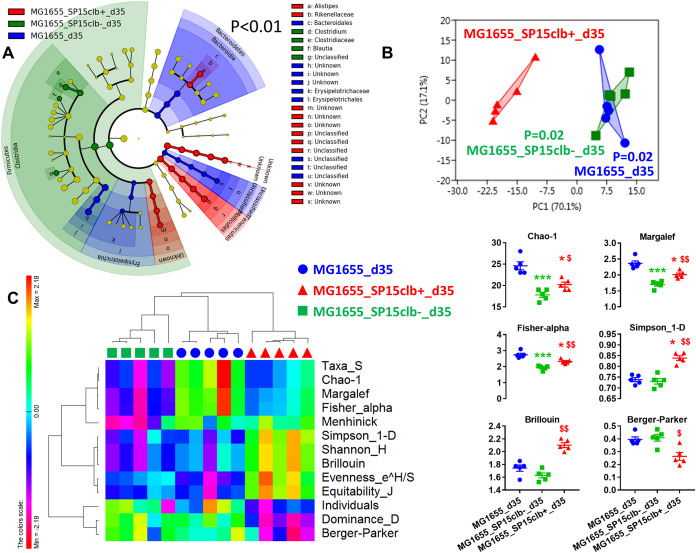
Cecum microbiota of mice aged 35 days following colonization with genotoxic or nongenotoxic E. coli. (A) Cladogram showing bacterial taxa significantly (*P* < 0.01) higher in the group of mouse pups of the same color, according to the enterobacterial strain received at birth. (B) PCA showing clusters of groups according to the cecum microbiota (following the color code of the overall figure). The *P* values compare the value for the group compared to the value for the genotoxic group (red). (C) Heat-map based on a Pearson distance and a complete linkage drawn with the PermutMatrix software (29) showing multiple diversity indices and dot-plot representation of the Simpson, Chao-1, Margalef, and Fisher-alpha indices on the right side. *n* = 5. *, *P* < 0.05; ***, *P* < 0.001 versus control group, Kruskal-Wallis test followed by a two-stage linear step-up procedure of Benjamini, Krieger, and Yekutieli to correct for multiple comparisons by controlling the false-discovery rate (<0.05). ^$^, *P* < 0.05; ^$$^, *P* < 0.01 between SP15clb- and SP15clb+ groups, Mann-Whitney test.

**FIG 5:**
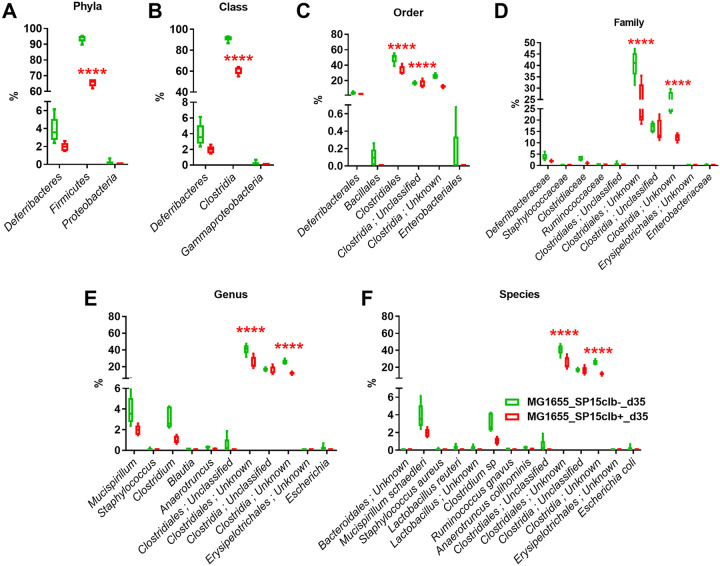
Bacterial taxon reduction following colonization at day 35 with genotoxic or nongenotoxic E. coli. (A to F) Phylum to species histogram representation. *n* = 5. ****, *P* < 0.0001, two-way ANOVA followed by a two-stage linear step-up procedure of Benjamini, Krieger, and Yekutieli to correct for multiple comparisons by controlling the false-discovery rate (<0.05).

**FIG 6:**
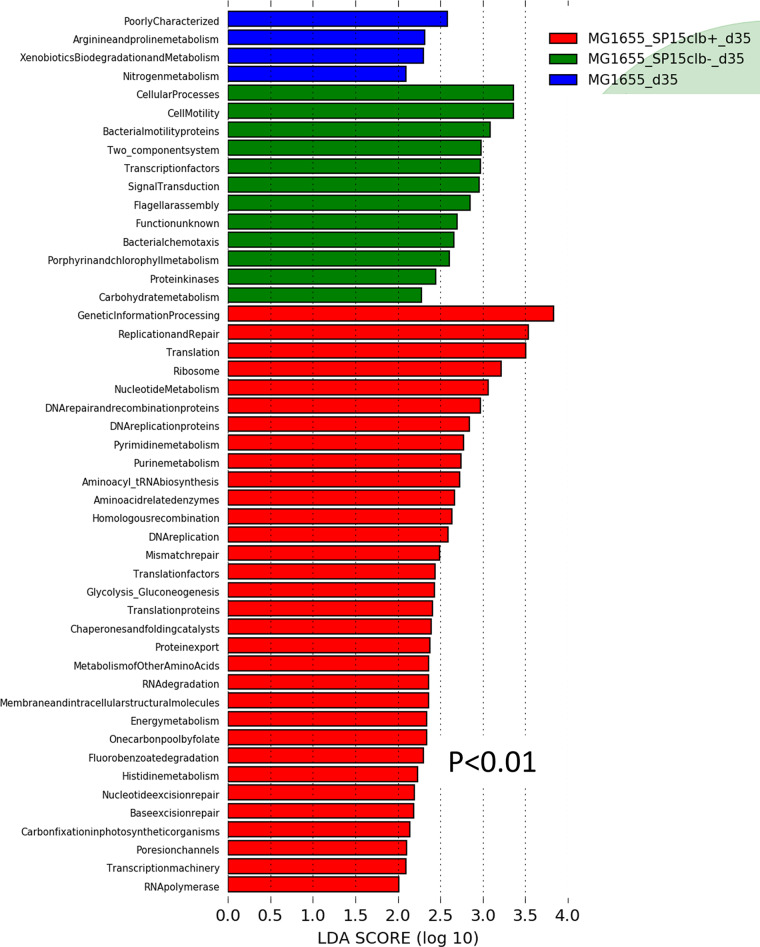
Cecum microbiome of mice aged 35 days following colonization with genotoxic or nongenotoxic E. coli. List of the bacterial functions enriched in each group and the related linear discriminant analysis (LDA) score (*P* < 0.01). *n* = 5.

Then, to investigate whether the early impact of colibactin had long-lasting consequences on gut microbiota composition, we analyzed the overall putative changes in the gut microbiota of mice 35 days after birth. Unlike what observed above at day 15, coinfection with the nongenotoxic E. coli SP15clb- strain was associated with a higher relative abundance of *Firmicutes*. In contrast, the coinfection with the genotoxic SP15clb+ strain was associated with a higher relative abundance of the genus *Alistipes* and family *Rikenellaceae* (Fig. 4A). A principal-component analysis (PCA) showed a complete separation between the gut microbiota profile of mice cocolonized with the genotoxic E. coli SP15clb+ strain compared to the other groups of mice (Fig. 4B). In addition, the calculation of several diversity indices showed a precise cluster separation among the three gut microbiota profiles, with a generally significantly higher diversity induced by the genotoxic strain compared to the nongenotoxic one, except for the Berger-Parker index (Fig. 4C). As for the reduction in microbial taxa, the phylum *Firmicutes* and all the other related taxa displayed a significantly lower abundance in mice colonized with the genotoxic E. coli SP15clb+ strain compared to the nongenotoxic E. coli SP15clb- strain (Fig. 5A to F). Next, we analyzed the gut microbiome by performing a functional analysis based on PICRUSt (18). As reported in Fig. 6, we identified microbial pathways significantly enriched in all three groups. In detail, microbial pathways related to replication and repair, DNA repair and recombination, proteins, and DNA replication, among all the pathways identified, were found significantly enriched in the mice cocolonized with the genotoxic E. coli SP15clb+ strain.

Overall, these data show that 35 days after birth of the mice, E. coli genotoxic activity profoundly affects the gut microbiota of the mouse pups at both a taxonomical and functional level and that it exerts an interspecies impact on the gut microbiota.

### Evolution of gut microbiota and microbiome following intestinal colonization by genotoxic or nongenotoxic E. coli SP15 strain in mice.

Next, we evaluated the effects of the infection with the E. coli strains reported above on the evolution of gut microbiota and microbiome by comparing the two time points at day 15 and day 35 after birth of the mice. On a taxonomical level, mice that received E. coli MG1655 strain displayed increased abundance of the family *Lachnospiraceae* on day 35 after birth (Fig. S3A); in terms of overall diversity, the infection with E. coli MG1655 did not induce a net separation between the two time points of 15 and 35 days (Fig. S3B), albeit some microbial functions were found significantly enriched (Fig. S3C). Mice that received the nongenotoxic E. coli SP15clb- strain displayed increased abundance of the phyla *Firmicutes* and *Deferribacteres* at day 35 after birth (Fig. S4A). The overall microbial diversity between the two time points of day 15 and day 35 was not affected (Fig. S4B), albeit some microbial functions were found significantly enriched (Fig. S4C). In contrast, mice that received the genotoxic E. coli SP15clb+ strain displayed increased levels of the phyla *Deferribacteres* and *Tenericutes* on day 35 after birth (Fig. S5A) and a net microbial diversity separation between the two time points of day 15 and day 35, with cellular processes related to signaling as the identified microbial function found significantly enriched (Fig. S5C). Overall, these data show that the genotoxic E. coli SP15clb+ strain affected the gut microbiota diversity to a greater extent than the other E. coli strains.

10.1128/mSphere.00589-20.3 FIG S3 Fifteen to 35 days after birth, cecum microbiota evolution of mouse pups which received MG1655 E. coli. (A) Cladogram showing bacterial taxa significantly (*P* < 0.01) higher in the group of mouse pups of the same color. (B) Heat-map based on a Pearson distance and a complete linkage drawn with the PermutMatrix software (29) showing multiple diversity indices. (C) LDA score of the bacterial functions significantly (*P* < 0.01) higher in the group of mouse pups of the same color. The threshold on the logarithmic LDA score for discriminative features was set at 3.0 (instead of 2.0). *n* = 5 per group. Download FIG S3, TIF file, 1.4 MB. Copyright © 2020 Tronnet et al. 2020 Tronnet et al. This content is distributed under the terms of the Creative Commons Attribution 4.0 International license.

10.1128/mSphere.00589-20.4 FIG S4 Fifteen to 35 days after birth, cecum microbiota evolution of mice which received MG1655 and E. coli SP15clb- strains. (A) Cladogram showing bacterial taxa significantly (*P* < 0.01) higher in the group of mouse pups of the same color. (B) Heat-map based on a Pearson distance and a complete linkage drawn with the PermutMatrix software (29) showing multiple diversity indices. (C) LDA score of the bacterial functions significantly (*P* < 0.01) higher in the group of mouse pups of the same color. The threshold on the logarithmic LDA score for discriminative features was set at 3.0 (instead of 2.0). *n* = 5 per group. Download FIG S4, TIF file, 1.9 MB. Copyright © 2020 Tronnet et al. 2020 Tronnet et al. This content is distributed under the terms of the Creative Commons Attribution 4.0 International license.

10.1128/mSphere.00589-20.5 FIG S5 Fifteen to 35 days after birth, cecum microbiota evolution of mice which received MG1655 and E. coli SP15clb+ strains. (A) Cladogram showing bacterial taxa significantly (*P* < 0.01) higher in the group of mouse pups of the same color. (B) Heat-map based on a Pearson distance and a complete linkage drawn with the PermutMatrix software (29) showing multiple diversity indices. (C) LDA score of the bacterial functions significantly (*P* < 0.01) higher in the group of mouse pups of the same color. The threshold on the logarithmic LDA score for discriminative features was set at 3.0 (instead of 2.0). *n* = 5 per group. Download FIG S5, TIF file, 1.4 MB. Copyright © 2020 Tronnet et al. 2020 Tronnet et al. This content is distributed under the terms of the Creative Commons Attribution 4.0 International license.

## DISCUSSION

In this study, we report the effects of the genotoxin colibactin on both gut microbiota and microbiome in mouse pups whose mothers had been colonized with either a genotoxic or nongenotoxic E. coli strain in combination with a nongenotoxic E. coli control strain. We adopted this protocol to monitor the natural mother-to-pup vertical transmission, which is among the strongest factors for E. coli dissemination in further generations (16). The genotoxin colibactin was demonstrated to act on eukaryotic cells by affecting DNA stability (7, 8) and to affect the host at multiple levels (9–13). These effects are likely to be blunted by an efficient intestinal mucous barrier, since an adherent mucous layer on epithelial cells was shown to dampen colibactin-induced DNA double-strand breaks *in vitro* (19).

It was recently reported that another genotoxin, the cytolethal distending toxin produced by the human clinical isolate Campylobacter jejuni 81-176, may affect both the microbial composition and gene expression profile of the gut microbiota of 1% dextran sulfate sodium-fed germfree (GF) *Apc^Min/+^* mice colonized with C. jejuni 81-176 (20). Beyond this evidence, we investigated whether colibactin may also exert its action against other bacteria of the gut microbiota, which was not known. Our data show a time-dependent double targeting action on gut microbes. (i) Fifteen days after birth, the maternally acquired E. coli genotoxic strain appears to exert an intraspecies effect on gut microbes by targeting bacteria from taxa belonging to the phylum *Proteobacteria*, to which E. coli belongs. (ii) Then, 35 days after birth, the action of colibactin appears directed against bacteria from *Proteobacteria*-unrelated taxa of the gut microbiota, such as those belonging to the phylum *Firmicutes*, showing an interspecies effect. Therefore, our data showing that colibactin may also target the gut microbiota may sustain the putative antibiotic activity of colibactin (17), which may first be devoted to help genotoxic bacteria creating their own niche. Then, once the niche was created, the genotoxic activity of bacteria may help them expand their niche by targeting gut microbes of unrelated taxa. This interpretation is also supported by the effects of colibactin we observed on the gut microbiome at day 35 after birth, where microbial pathways related to DNA repair were the most affected. This evidence is in line with the capacity of colibactin to affect DNA stability (7, 8) and thus induce the DNA repair machinery.

In terms of the evolution of the gut microbiota, the genotoxin colibactin strongly changed the overall microbial diversity from day 15 to day 35 in mice which received the genotoxic E. coli strain, compared to mice which received the nongenotoxic E. coli strain.

Our data suggest that, in mice, the bacterial genotoxin colibactin may exert its activity even against the gut microbiota, beyond its known effects on the host (7, 9). To ascribe only to colibactin the effects we wanted to study, we used two isogenic mutants differing only in the colibactin-maturating gene *clbP* (21). Despite this strategy, we cannot totally exclude the possibility that other factors such as bacterial growth, replication, stress response to other stressors and other secondary metabolites may account for the observed results that, hence, may not specifically and unambiguously implicate colibactin itself. However, we recently demonstrated (15) that (i) the mutant of the *clbP* catalytic site used in our study does not affect siderophe-microcin production and (ii) the SP15 strain used does not produce siderophore-microcin, because is deprived of Mcc genes. Therefore, at least siderophore-microcin production appears not to account in this context. Furthermore, the effects of the colibactin-producing strain may be indirect, due to the impact on the host intestinal mucosa, such as increased reactive oxygen species (ROS) production (22), which, in turn, may alter both composition and function of gut microbiota.

Overall, the implication of all of this evidence may help in understanding the benefits that the acquisition of genotoxicity may provide to *pks*^+^ bacteria, such as the establishing and expanding of their own niche within the complex intestinal microbial ecosystem.

## MATERIALS AND METHODS

### Animal model and tissue collection.

The animal model used in this study was already published (12). Briefly, primiparous timed pregnant Swiss CD1 mice were purchased from Janvier Labs, housed separately under specific-pathogen-free conditions with access to food and water supplemented with streptomycin (5g/liter) *ad libitum*. Pregnant SWISS CD1 females were inoculated once with a total of 10^9^ bacteria (E. coli MG1655 alone or in combination with SP15_clb+ or SP15_clb- strain, each at 5 × 10^8^ bacteria to have a total of 10^9^ bacteria) by intragastric gavage the day before parturition. Mice were sacrificed in a fed state by cervical dislocation; then, cecal tissues were collected and snap-frozen in liquid nitrogen. All animal experimental procedures were approved by the local ethical committee (protocol CE2017031317082461V3) of Purpan University Hospital (Toulouse, France). All experiments were performed in accordance with relevant guidelines and regulations.

### Strain construction.

E. coli strain SP15 is an extraintestinal pathogenic E. coli (ExPEC) strain of serotype O18:K1 isolated from a patient with neonatal meningitis (23). Strain SP15 harbors the *pks* island and was previously shown to produce colibactin (24). Inactivation of the gene *clbP* (21) was engineered by using the lambda Red recombinase method (25) using primers clbP-P1 and clbP-P2 (see Fig. S2 in the supplemental material) followed by excision of the kanamycin resistance cassette, as previously described (25), to produce strain SP15Δclb-. The functional wild-type *clbP* gene and the *clbP* gene that was site directed mutagenized to inactivate the catalytic site of the ClbP enzyme were PCR amplified from vectors pBRSK-clbP and pBRSK-clbP-S95A (21), respectively, using primers clbP-F-Bam and pBRSK-F-Bam (Fig. S2). The resulting PCR products were restricted by BamHI and cloned into vector pCM17 (26) digested by BamHI. The resulting plasmids pCM17-clbP and pCM17-clbP-S95A were transformed into strain SP15Δclb-. The resulting strains SP15clb+ and SP15clb- were demonstrated to produce colibactin and not produce colibactin, respectively.

### Taxonomic analysis of the cecum microbiota by MiSeq.

Total DNA was extracted (27) from ceca at both day 15 and day 35 after birth of the mice. The 16S rRNA gene V3-V4 regions were targeted by the 357wf-785R primers and analyzed by MiSeq at RTL Genomics (Lubbock, TX, USA). An average of 19,825 sequences was generated per sample. A complete description of the applied bioinformatic filters is available at https://www.rtlgenomics.com/docs/Data_Analysis_Methodology.pdf. Cladograms were drawn by the Huttenhower Galaxy web application (http://huttenhower.sph.harvard.edu/galaxy/) via the linear discriminant analysis (LDA) effect size (LEfSe) algorithm (28). LDA scores have been calculated as specified at https://huttenhower.sph.harvard.edu/galaxy/, tool LEfSe, point B “LDA Effect Size (LEfSe).”

### Statistical analysis.

Results are presented as means ± standard errors of the means (SEM). Statistical analyses were performed by two-way analysis of variance (ANOVA) followed by a two-stage linear step-up procedure of Benjamini, Krieger, and Yekutieli to correct for multiple comparisons by controlling the false-discovery rate (<0.05) or Kruskal-Wallis test plus a two-stage step-up method of Benjamini, Krieger, and Yekutieli correction for multiple comparisons by controlling the false-discovery rate (<0.05) or Mann-Whitney test, as indicated in the figure legend, by using GraphPad Prism version 7.00 for Windows Vista (GraphPad Software, San Diego, CA). Values were considered significant when the *P* value was <0.05 or as reported after corrections. For cladograms, the alpha value for the factorial Kruskal-Wallis test among classes and the alpha value for the pairwise Wilcoxon test between subclasses have been changed to *P* < 0.01, as shown on figures. Principal-component analyses were drawn, and diversity indices were calculated with the software PAST 4 (https://folk.uio.no/ohammer/past/). Heat-maps based on a Pearson distance and a complete linkage were drawn with the PermutMatrix software (29). Data shown on heat-maps have been centered reduced to improve the color readout.

### Availability of data and materials.

All data are available in the main text or the supplemental material and via the following repositories: Sequence Read Archive (SRA) database https://submit.ncbi.nlm.nih.gov/subs/sra/ with the assigned identifier PRJNA593936.

## References

[1] Serino M. 2018. Molecular paths linking metabolic diseases, gut microbiota dysbiosis and enterobacteria infections. J Mol Biol 430:581–590. doi:10.1016/j.jmb.2018.01.010. 29374557

[2] Agus A, Denizot J, Thevenot J, Martinez-Medina M, Massier S, Sauvanet P, Bernalier-Donadille A, Denis S, Hofman P, Bonnet R, Billard E, Barnich N. 2016. Western diet induces a shift in microbiota composition enhancing susceptibility to adherent-invasive E. coli infection and intestinal inflammation. Sci Rep 6:19032. doi:10.1038/srep19032. 26742586 PMC4705701

[3] Secher T, Brehin C, Oswald E. 2016. Early settlers: which E. coli strains do you not want at birth? Am J Physiol Gastrointest Liver Physiol 311:G123–G129. doi:10.1152/ajpgi.00091.2016. 27288422

[4] Croxen MA, Finlay BB. 2010. Molecular mechanisms of Escherichia coli pathogenicity. Nat Rev Microbiol 8:26–38. doi:10.1038/nrmicro2265. 19966814

[5] Tenaillon O, Skurnik D, Picard B, Denamur E. 2010. The population genetics of commensal Escherichia coli. Nat Rev Microbiol 8:207–217. doi:10.1038/nrmicro2298. 20157339

[6] Bach JF. 2002. The effect of infections on susceptibility to autoimmune and allergic diseases. N Engl J Med 347:911–920. doi:10.1056/NEJMra020100. 12239261

[7] Nougayrede JP, Homburg S, Taieb F, Boury M, Brzuszkiewicz E, Gottschalk G, Buchrieser C, Hacker J, Dobrindt U, Oswald E. 2006. Escherichia coli induces DNA double-strand breaks in eukaryotic cells. Science 313:848–851. doi:10.1126/science.1127059. 16902142

[8] Bossuet-Greif N, Vignard J, Taieb F, Mirey G, Dubois D, Petit C, Oswald E, Nougayrède J-P. 2018. The colibactin genotoxin generates DNA interstrand cross-links in infected cells. mBio 9:e02393-17. doi:10.1128/mBio.02393-17. 29559578 PMC5874909

[9] Cougnoux A, Dalmasso G, Martinez R, Buc E, Delmas J, Gibold L, Sauvanet P, Darcha C, Dechelotte P, Bonnet M, Pezet D, Wodrich H, Darfeuille-Michaud A, Bonnet R. 2014. Bacterial genotoxin colibactin promotes colon tumour growth by inducing a senescence-associated secretory phenotype. Gut 63:1932–1942. doi:10.1136/gutjnl-2013-305257. 24658599

[10] Secher T, Samba-Louaka A, Oswald E, Nougayrède J-P. 2013. Escherichia coli producing colibactin triggers premature and transmissible senescence in mammalian cells. PLoS One 8:e77157. doi:10.1371/journal.pone.0077157. 24116215 PMC3792898

[11] Marcq I, Martin P, Payros D, Cuevas-Ramos G, Boury M, Watrin C, Nougayrede JP, Olier M, Oswald E. 2014. The genotoxin colibactin exacerbates lymphopenia and decreases survival rate in mice infected with septicemic Escherichia coli. J Infect Dis 210:285–294. doi:10.1093/infdis/jiu071. 24489107

[12] Payros D, Secher T, Boury M, Brehin C, Menard S, Salvador-Cartier C, Cuevas-Ramos G, Watrin C, Marcq I, Nougayrede JP, Dubois D, Bedu A, Garnier F, Clermont O, Denamur E, Plaisancie P, Theodorou V, Fioramonti J, Olier M, Oswald E. 2014. Maternally acquired genotoxic Escherichia coli alters offspring’s intestinal homeostasis. Gut Microbes 5:313–325. doi:10.4161/gmic.28932. 24971581 PMC4153768

[13] Dalmasso G, Cougnoux A, Delmas J, Darfeuille-Michaud A, Bonnet R. 2014. The bacterial genotoxin colibactin promotes colon tumor growth by modifying the tumor microenvironment. Gut Microbes 5:675–680. doi:10.4161/19490976.2014.969989. 25483338 PMC4615906

[14] Perez-Berezo T, Pujo J, Martin P, Le Faouder P, Galano JM, Guy A, Knauf C, Tabet JC, Tronnet S, Barreau F, Heuillet M, Dietrich G, Bertrand-Michel J, Durand T, Oswald E, Cenac N. 2017. Identification of an analgesic lipopeptide produced by the probiotic Escherichia coli strain Nissle 1917. Nat Commun 8:1314. doi:10.1038/s41467-017-01403-9. 29101366 PMC5670229

[15] Massip C, Branchu P, Bossuet-Greif N, Chagneau CV, Gaillard D, Martin P, Boury M, Sécher T, Dubois D, Nougayrède J-P, Oswald E. 2019. Deciphering the interplay between the genotoxic and probiotic activities of Escherichia coli Nissle 1917. PLoS Pathog 15:e1008029. doi:10.1371/journal.ppat.1008029. 31545853 PMC6776366

[16] Mackie RI, Sghir A, Gaskins HR. 1999. Developmental microbial ecology of the neonatal gastrointestinal tract. Am J Clin Nutr 69:1035S–1045S. doi:10.1093/ajcn/69.5.1035s. 10232646

[17] Fais T, Cougnoux A, Dalmasso G, Laurent F, Delmas J, Bonnet R. 2016. Antibiotic activity of Escherichia coli against multiresistant Staphylococcus aureus. Antimicrob Agents Chemother 60:6986–6988. doi:10.1128/AAC.00130-16. 27600034 PMC5075125

[18] Langille MG, Zaneveld J, Caporaso JG, McDonald D, Knights D, Reyes JA, Clemente JC, Burkepile DE, Vega Thurber RL, Knight R, Beiko RG, Huttenhower C. 2013. Predictive functional profiling of microbial communities using 16S rRNA marker gene sequences. Nat Biotechnol 31:814–821. doi:10.1038/nbt.2676. 23975157 PMC3819121

[19] Reuter C, Alzheimer M, Walles H, Oelschlaeger TA. 2018. An adherent mucus layer attenuates the genotoxic effect of colibactin. Cell Microbiol 20:10.1111/cmi.12812. doi:10.1111/cmi.12812. 29156489

[20] He Z, Gharaibeh RZ, Newsome RC, Pope JL, Dougherty MW, Tomkovich S, Pons B, Mirey G, Vignard J, Hendrixson DR, Jobin C. 2019. Campylobacter jejuni promotes colorectal tumorigenesis through the action of cytolethal distending toxin. Gut 68:289–300. doi:10.1136/gutjnl-2018-317200. 30377189 PMC6352414

[21] Dubois D, Baron O, Cougnoux A, Delmas J, Pradel N, Boury M, Bouchon B, Bringer MA, Nougayrede JP, Oswald E, Bonnet R. 2011. ClbP is a prototype of a peptidase subgroup involved in biosynthesis of nonribosomal peptides. J Biol Chem 286:35562–35570. doi:10.1074/jbc.M111.221960. 21795676 PMC3195562

[22] Veziant J, Gagniere J, Jouberton E, Bonnin V, Sauvanet P, Pezet D, Barnich N, Miot-Noirault E, Bonnet M. 2016. Association of colorectal cancer with pathogenic Escherichia coli: focus on mechanisms using optical imaging. World J Clin Oncol 7:293–301. doi:10.5306/wjco.v7.i3.293. 27298769 PMC4896897

[23] Johnson JR, Oswald E, O’Bryan TT, Kuskowski MA, Spanjaard L. 2002. Phylogenetic distribution of virulence-associated genes among Escherichia coli isolates associated with neonatal bacterial meningitis in the Netherlands. J Infect Dis 185:774–784. doi:10.1086/339343. 11920295

[24] Martin P, Marcq I, Magistro G, Penary M, Garcie C, Payros D, Boury M, Olier M, Nougayrede JP, Audebert M, Chalut C, Schubert S, Oswald E. 2013. Interplay between siderophores and colibactin genotoxin biosynthetic pathways in Escherichia coli. PLoS Pathog 9:e1003437. doi:10.1371/journal.ppat.1003437. 23853582 PMC3708854

[25] Datsenko KA, Wanner BL. 2000. One-step inactivation of chromosomal genes in Escherichia coli K-12 using PCR products. Proc Natl Acad Sci U S A 97:6640–6645. doi:10.1073/pnas.120163297. 10829079 PMC18686

[26] Morin CE, Kaper JB. 2009. Use of stabilized luciferase-expressing plasmids to examine in vivo-induced promoters in the Vibrio cholerae vaccine strain CVD 103-HgR. FEMS Immunol Med Microbiol 57:69–79. doi:10.1111/j.1574-695X.2009.00580.x. 19678844 PMC2906245

[27] Serino M, Luche E, Gres S, Baylac A, Berge M, Cenac C, Waget A, Klopp P, Iacovoni J, Klopp C, Mariette J, Bouchez O, Lluch J, Ouarne F, Monsan P, Valet P, Roques C, Amar J, Bouloumie A, Theodorou V, Burcelin R. 2012. Metabolic adaptation to a high-fat diet is associated with a change in the gut microbiota. Gut 61:543–553. doi:10.1136/gutjnl-2011-301012. 22110050 PMC3292714

[28] Segata N, Izard J, Waldron L, Gevers D, Miropolsky L, Garrett WS, Huttenhower C. 2011. Metagenomic biomarker discovery and explanation. Genome Biol 12:R60. doi:10.1186/gb-2011-12-6-r60. 21702898 PMC3218848

[29] Caraux G, Pinloche S. 2005. PermutMatrix: a graphical environment to arrange gene expression profiles in optimal linear order. Bioinformatics 21:1280–1281. doi:10.1093/bioinformatics/bti141. 15546938

