# Supplementary figures and images for: The Genotoxin Colibactin Shapes Gut Microbiota in Mice
Source: mSphere. 2020 Jul 1;5(4):e00589-20. doi: 10.1128/mSphere.00589-20 (PMC7333578; doi:10.1128/mSphere.00589-20)

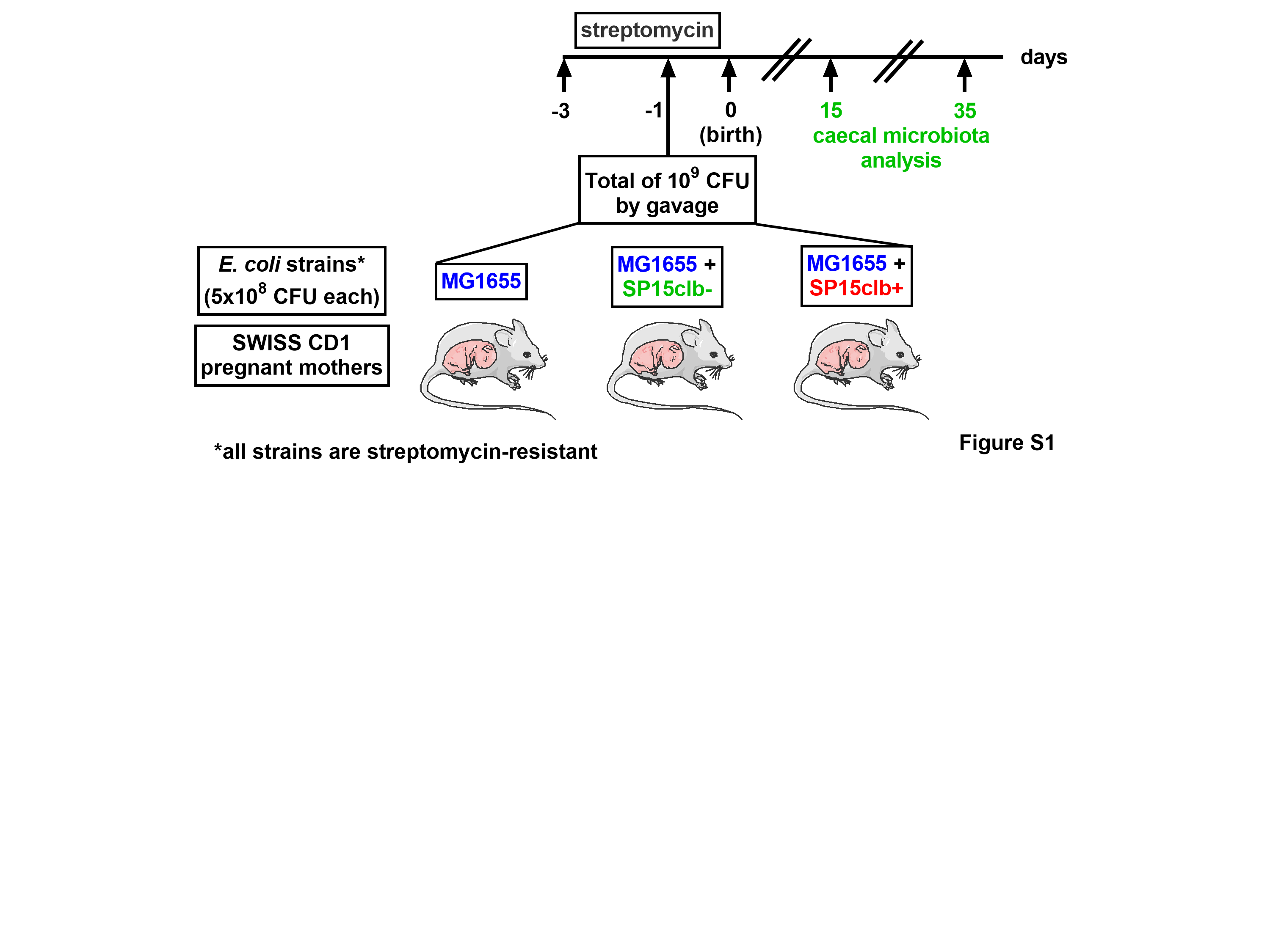

Supplement: FIG S1 [file mSphere.00589-20-sf001.tif]

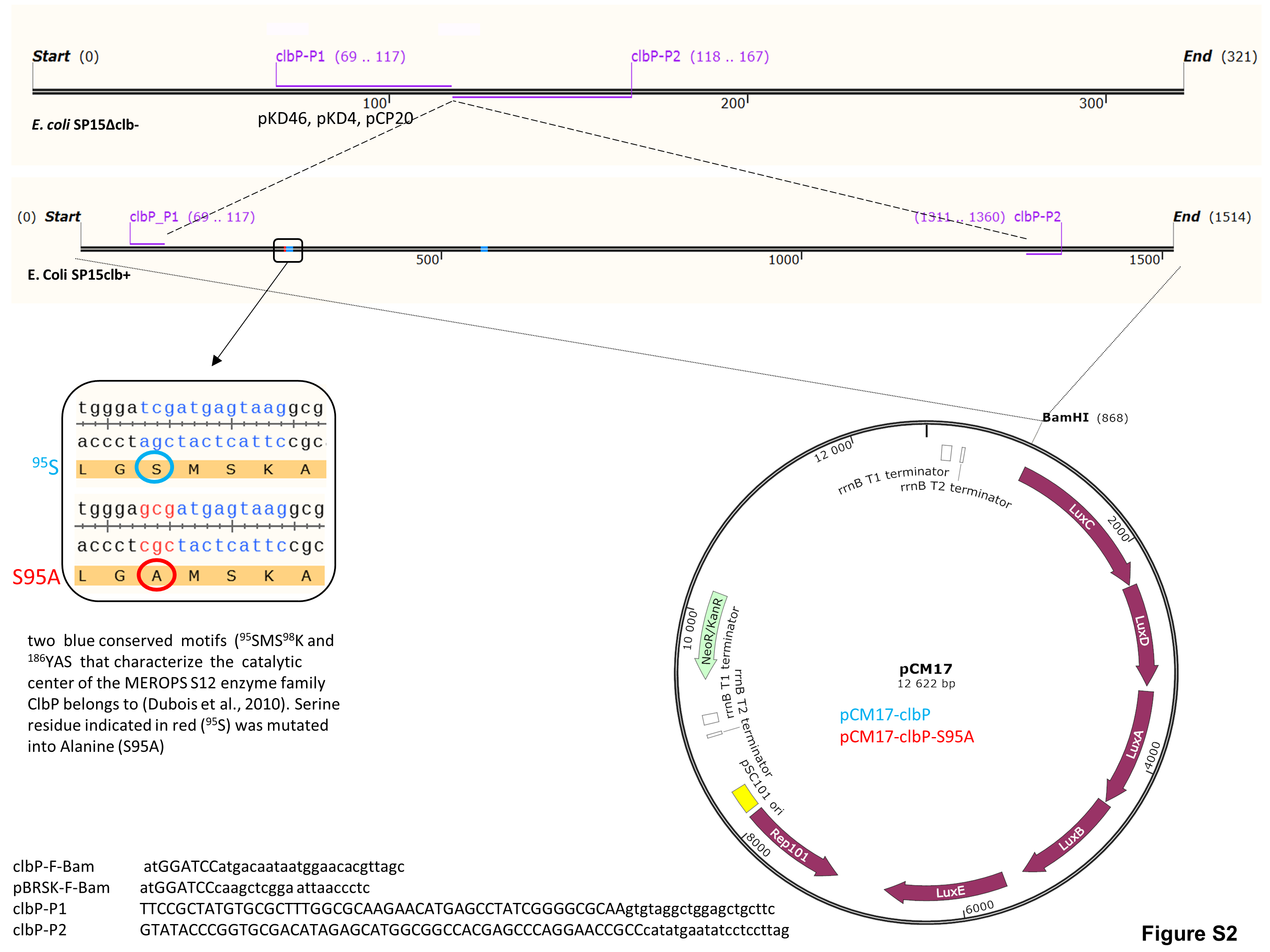

Supplement: FIG S2 [file mSphere.00589-20-sf002.tif]

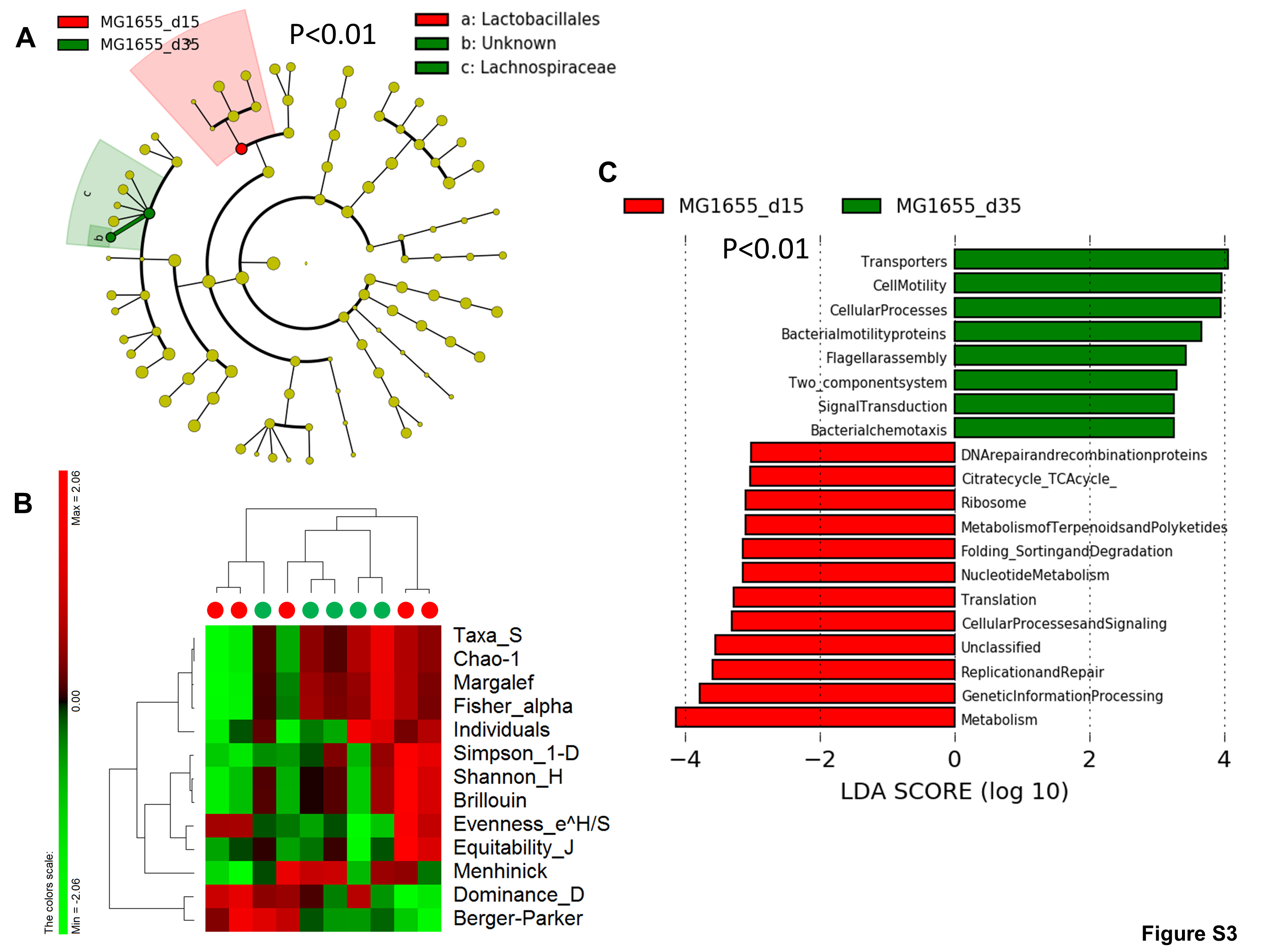

Supplement: FIG S3 [file mSphere.00589-20-sf003.tif]

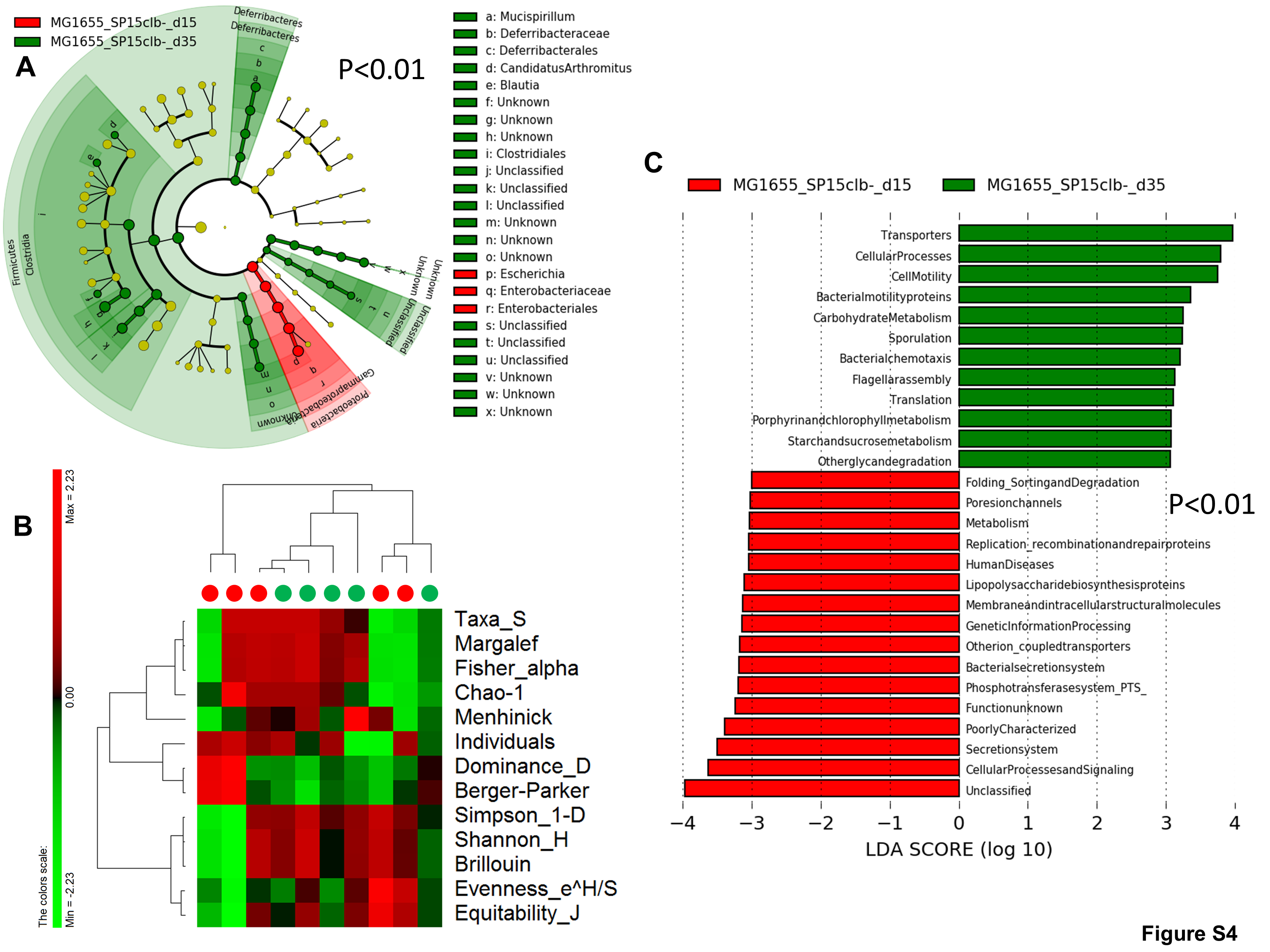

Supplement: FIG S4 [file mSphere.00589-20-sf004.tif]

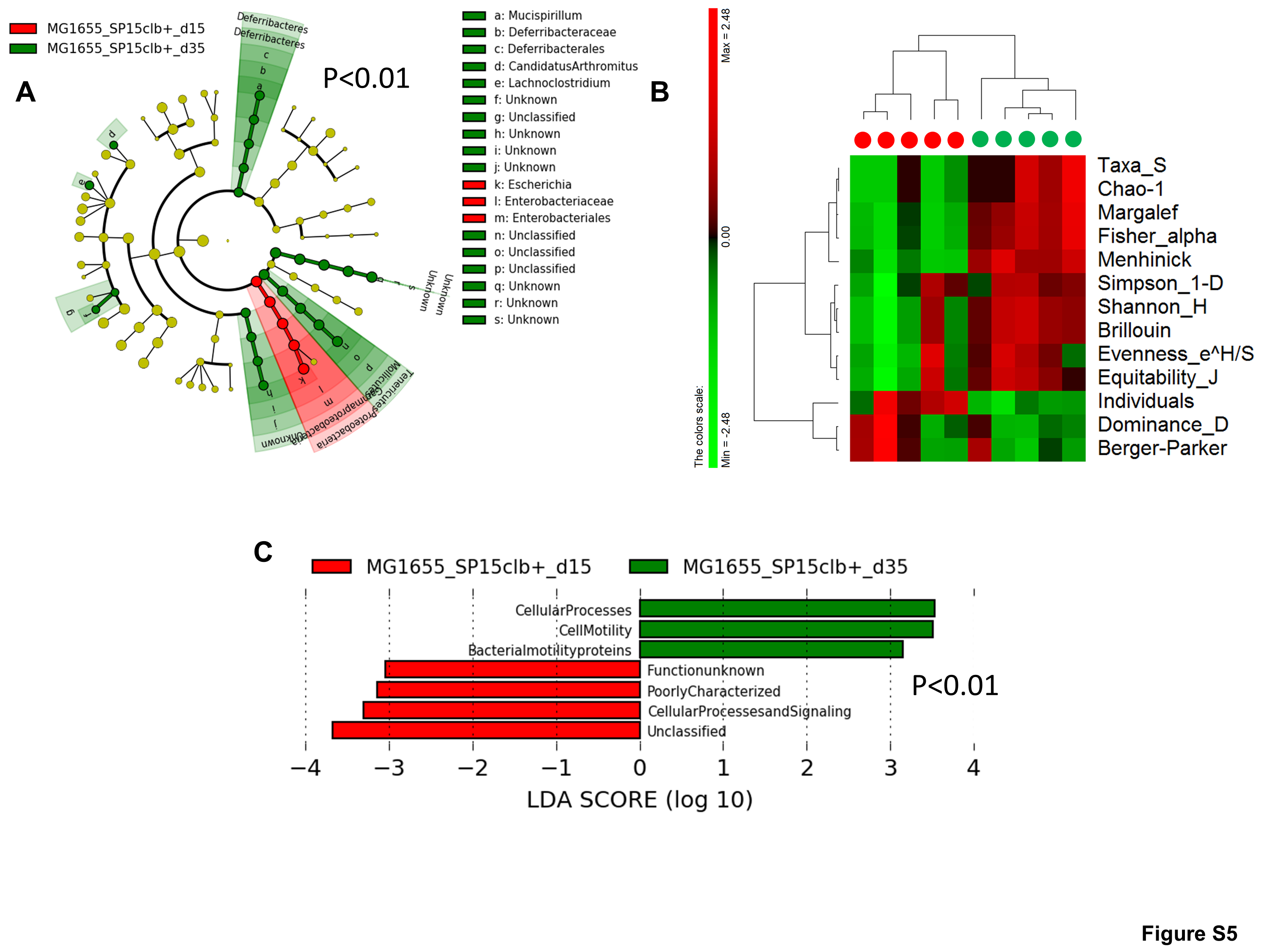

Supplement: FIG S5 [file mSphere.00589-20-sf005.tif]
